# Fibromodulin deficiency reduces collagen structural network but not glycosaminoglycan content in a syngeneic model of colon carcinoma

**DOI:** 10.1371/journal.pone.0182973

**Published:** 2017-08-21

**Authors:** P. Olof Olsson, Sebastian Kalamajski, Marco Maccarana, Åke Oldberg, Kristofer Rubin

**Affiliations:** 1 Department of Laboratory Medicine, Translational Cancer Research, Medicon Village, Lund University, SE,Lund, Sweden; 2 Department of Medical Biochemistry and Microbiology, SciLife Laboratories, Uppsala University, BMC, SE,Uppsala, Sweden; 3 Department of Experimental Medicine, Matrix Biology, SE, Lund, Sweden; University of Patras, GREECE

## Abstract

Tumor barrier function in carcinoma represents a major challenge to treatment and is therefore an attractive target for increasing drug delivery. Variables related to tumor barrier include aberrant blood vessels, high interstitial fluid pressure, and the composition and structure of the extracellular matrix. One of the proteins associated with dense extracellular matrices is fibromodulin, a collagen fibrillogenesis modulator expressed in tumor stroma but scarce in normal loose connective tissues. Here, we investigated the effects of fibromodulin on stroma ECM in a syngeneic murine colon carcinoma model. We show that fibromodulin deficiency decreased collagen fibril thickness but glycosaminoglycan content and composition were unchanged. Furthermore, vascular density, pericyte coverage and macrophage amount were unaffected. Fibromodulin can therefore be a unique effector of dense collagen matrix assembly in tumor stroma and, without affecting other major matrix components or the cellular composition, can function as a main agent in tumor barrier function.

## Introduction

Carcinoma stroma features a dense and dysfunctional extracellular matrix (ECM), which together with aberrant vessels and an underdeveloped lymphatic system, results in elevated interstitial fluid pressure (IFP) and barrier to treatment [1]. In carcinoma, a dense stromal ECM can form a functional barrier for fluid transport, promote malignant progression, and impair blood flow [2–5]. These abnormalities negatively affect the outcome of chemo- and radio-therapeutic treatments [6–9]. Treatment with the tyrosine kinase inhibitor Imatinib (STI-571) reduces IFP, increases extracellular volume (ECV), and increases tumor perfusability, as evidenced by increased fluid transport and tumor tissue oxygenation in ECM-rich carcinomas [10–14]. Furthermore, a specific inhibitor of TGF- β1 and - β3 lowers IFP in experimental carcinoma [15, 16].

Fibromodulin is an extracellular small leucine-rich proteoglycan (SLRP) glycosylated with keratan sulfate or polylactosamine chains [17]. It interacts with type I and II collagens and, together with other SLRPs, modulates collagen fibrillogenesis [18]. It is, however, not known if fibromodulin affects the structure and amounts of the hyaluronan and proteoglycan-rich ground substance in connective tissue. Fibromodulin deficiency in mice does not impart any overt phenotypic difference, apart from decreased tendon stiffness [19–21]. Fibromodulin-deficiency reduces IFP and increases ECV in experimental carcinoma and leads to decreased collagen fibril thickness in experimental carcinoma and in tail tendons [19, 22]. Furthermore, expression of fibromodulin decreases in experimental carcinomas treated with a specific inhibitor of TGF-ß1 and -ß3, which correlates with decreased collagen fibril thickness [22]. Similarly, treatment of experimental carcinoma with Imatinib reduces collagen fibril thickness [12].

In ECM, interstitial fluid flow depends on the densities and structures of the collagen fiber network, on the microfibrillar network, and on the glycosaminoglycan-rich ground substance [23, 24]. We have previously shown that fibromodulin modulates collagen fibers in tumor extracellular environment [22]. Since the glycosaminoglycan content and quality is an important regulator of interstitial fluid volume [23] we investigated whether fibromodulin deficiency not only affects the stromal collagen network but also affects glycosaminoglycans in experimental carcinoma.

## Materials and methods

### Tumor model

The local *Fmod*-/- C57Bl6 mouse line previously described [19] was bred for six generations with C57Bl6 from Jackson laboratory (Bar Harbor, ME USA). All mice used in the experiments were generated by breeding wild-type and *Fmod*-/- sibling pairs. These breeding groups were expanded further by one generation, resulting in all used mice being separated by at most two generations.

C38 mouse colon carcinoma cells, also referred to as Colon38, C38 or MC38, syngeneic to the C57Bl6 mouse line [25, 26], were selected by tumor passage in animals. C38 clone exhibiting colonial growth, denoted OOC38 was expanded and became the base for all tumor generation in this study. The cell line was shown to not have undergone epithelial-to-mesenchymal transition as judged from gene expression profiles of microfilament and ECM proteins (data not shown).

Human colon adenocarcinoma KAT-4/HT-29 (American Type Culture Collection, LGC Standards GmbH, Wesel Germany) were initially described as originating from a thyroid tumor [27]; however, a thyroid origin of the KAT-4 carcinoma was later questioned and the cells have been identified as originating from colorectal adenocarcinoma cell line HT-29 [12, 28].

5x10^6^ OOC38 cells were injected *s*.*c*. in the left flank of wild-type and *Fmod*-/- C57Bl6 mice and 2 x 10^6^ KAT-4 cells in 100 μL PBS were injected *s*.*c*. in the left flank of six- to eight-week old Fox Chase SCID mice (M&B, Ry, Denmark). Tumor growth was monitored with a caliper and external volumetric measurements of tumors were calculated by multiplying length, width and height. Mice were sacrificed using isofluorane when tumors reached an external size of around 1 cm^3^. No further treatment or ingress was performed. Mice were bred and maintained at the animal facility at Lund University. All animal experiments were approved by the local ethics committee at Lund University and performed according to the UKCCCR guidelines [29]

### Electron microscopy

OOC38-derived carcinoma from wild-type and *Fmod-/-* mice were fixed in 0.15 M sodium cacodylate-buffered 2.5% glutaraldehyde, post-fixed in 0.15 M sodium cacodylate-buffered 1% osmium tetraoxide, dehydrated in graded ethanol series, impregnated in acetone and embedded in epoxy resin. Ultra-thin sections were examined in a Tecnai Spirit BioTWIN transmission electron microscope (FEI company, OR, USA) and the micrographs were quantified with ImageJ software (NIH, MD, USA).

### Hydroxyproline determinations

KAT-4/HT-29 and OOC38 carcinomas were hydrolyzed in 6M HCl for 4 hours at 120°C at 2 atm pressure. Hydroxyproline content in the hydrolysates was determined as described [30].

### Glycosaminoglycan analyses

Carcinoma tissues were lyophilized. Glycosaminoglycan preparation, lyase treatment, fluorescence disaccharide labeling and separation were performed according to [31]. Briefly, tumors were protease- and DNAase digested, and GAGs were purified on anion-exchange chromatography. Then, 500ng GAGs, as estimated by the carbazol method, were subjected to degradation by chondroitinase ABC (Sigma) or by a mixture of heparinases (in-house preparation, purified from *E*.*coli* transformed with the pET-15b expression vector carrying heparinase I, or pET-19b expression vector carrying heparinase II or III; provided by prof. Jian Liu, University of North Carolina). Fluorophore-labeling of the resulting disaccharides was performed by 2-aminoacridone (AMAC, Sigma). Pre-column AMAC-labeled disaccharides were analyzed by HPLC as described previously [31]. Quantification was done by comparison to known weight of mock-treated standard disaccharides (Iduron, UK).

### RT-PCR and real-time qPCR

Total RNA was extracted from tumors (five biological replicates) using Trizol reagent (Thermofisher, Stockholm, Sweden). 500 ng RNA was used for reversed transcription using Superscript VILO (Thermofisher). Real-time qPCR was performed with Taqman probes, listed previously [12], using an Applied Biosystems 7300 detection system. Gene expression was normalized to the *Actb* transcript.

### Immunofluorescence

Tumors were snap-frozen at -80° C, embedded in OCT (Sakura, Torrence CA, USA) and sectioned. Frozen 10 μm sections were fixed in 4% paraformaldehyde (Merck, Darmstadt, Germany) or acetone (Sigma), blocked in 40% serum (20% Goat serum (Serotec, Oxford, UK) and 20% horse serum (SVA, Uppsala, Sweden) or pig serum from Chemicon (Temacula, CA, USA) and incubated with the following primary antibodies: monoclonal rat anti-mouse CD31 clones Mec 13.3 and 390 (BD, San Diego, CA, USA), monoclonal rat anti-mouse F4/80 (Serotec, Bio-Rad), polyclonal rabbit anti-mouse NG2 (Chemicon), and anti-mouse α-Smooth Muscle Actin (α-SMA) clone 1A4 FITC-conjugated (Sigma). The following secondary antibodies were used: FITC-conjugated goat anti-rat IgG and Texas Red-conjugated goat anti-rabbit (Vector laboratories, Burlingame, CA, USA). Exchange of primary and secondary antibodies for mouse or rabbit normal IgG or PBS was performed in all combinations necessary to establish the observed staining specificity. DAPI (4',6-diamidino-2-phenylindole) from Sigma was used for nuclear staining. Images were retrieved with a Nikon Eclipse 90i microscope (Nikon Instruments, Amstelveen, The Netherlands). Image analysis and pixel quantification was performed with Photoshop (Adobe, San José, CA, USA) and ImageJ (NIH) software, respectively. The mean percentage of pixels per image was calculated from several images per investigated tumor. Co-localization data is presented as the percentage of CD31-positive pixels that co-localized with NG2, and vice versa.

## Results

### Characteristics of OOC38 carcinoma grown in Fmod-/- and wild-type mice

**Fig 1:**
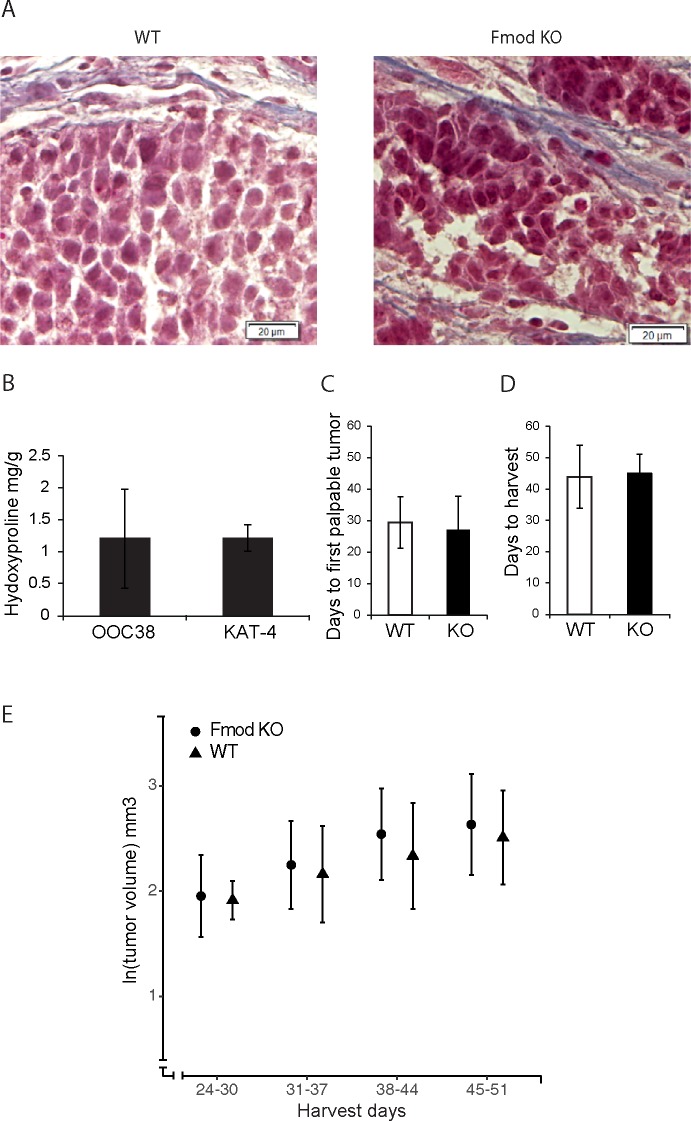
Properties of OOC38-derived carcinoma in wild-type and *Fmod-/-* mice. A) Masson Trichrome staining of OOC38 tumors grown in wild-type and *Fmod-/-* mice, depicting morphology and collagen fibers. B) Hydroxyproline content of OOC38 and KAT-4 tumors (WT n = 4, *Fmod*-/- n = 4), expressed as mg hydroxyproline/g tumor wet weight. C) Average number of days to first measurable tumor post cell inoculation (WT n = 18, *Fmod-/-* n = 32). D) Average number of days at which tumors reached an external measurement of 1 mm^3^ (WT n = 15, *Fmod*-/- n = 30). E) Ln values of externally measured tumor volumes (data points are means; error bars are standard deviations). Tumors were harvested within the indicated time frames (days post-injection: 24–30, 31–37, 38–44, 45–51). Number of tumors were, respectively: wild-type n = 8, 20, 33, 20; *Fmod -/-* n = 64, 51, 66, 47. No differences in tumor exponential growth were observed between WT and *Fmod*-/- (Student’s *t*-test, p>0.05).

Gross morphological staining of OOC38 carcinoma grown in wild-type and in *Fmod*-/- C57Bl6 mice did not reveal any noticeable difference in tumor appearance, *i*.*e*. in cancer cell distribution and density or in ECM localization (Fig 1A). Similar collagen quantities (hydroxyproline concentration) were present in OOC38 carcinoma and human colorectal KAT-4 carcinoma grown in wild-type mice (Fig 1B). We next quantified ECM-related gene transcripts by qPCR. No differences were detected in the levels of transcripts encoding collagens (Col1a1, Col3a1, Col6a1), collagen fibril maturation-related proteins (lumican, decorin), collagen crosslinking-related proteins (lysine oxidase, lysine hydroxylase 2, lysine oxidase-like 1 and 2), matrix metalloproteinases (Mmp—2 and -3), and the ECM-proteins fibrillin1 and periostin (Table 1). Furthermore, fibrin deposition, determined from immuno-stained carcinoma sections, was not different (wild-type, n = 12; *Fmod-/-*, n = 18). Fibrin-positive areas, in total areas of the investigated fields of vision, averaged 3.4% and 3.9% respectively (p = 0.7).

**Table 1:** Gene expression (mRNA) data represented as the percent (%) of carcinoma grown in *Fmod-/-* compared to wild type mice.

			WT	Fmod KO			
Gene	WT (%)	Student´s t test p value	dCt	Sd dCT	dCt	Sd dCT	n
*Col1a1*	95	0,88	3,76	1,05	3,84	0,42	5
*Col3a1*	136	0,41	6,81	0,80	6,36	0,83	5
*Lox*	91	0,78	8,70	0,70	8,84	0,80	5
*Plod2*	108	0,80	5,17	0,75	5,07	0,47	5
*Fbn1*	106	0,86	9,06	0,74	8,97	0,80	5
*Mmp2*	72	0,22	10,27	0,59	10,74	0,50	5
*Mmp3*	65	0,40	11,00	1,26	11,63	0,98	5
*Col6a1*	106	0,80	12,13	0,49	12,05	0,43	5
Fmod*	9	0,00071	14,86	1,33	18,33	0,60	5
*Lum*	130	0,55	13,68	0,89	13,30	1,01	5
*Dcn*	74	0,51	9,35	1,06	9,77	0,87	5
*Loxl1*	189	0,10	12,32	0,92	11,41	0,59	5
*Loxl2*	115	0,70	9,83	0,87	9,63	0,71	5
*Postn*	121	0,29	6,06	0,73	5,77	0,85	5

qPRC values reported as percent of WT and dCT. (n = 5+5)

*Fmod included as a verification of genotype/method

Growth characteristics of OOC38 carcinoma were similar in *Fmod*-/- and in wild-type mice. There was no difference in the lag phase, *i*.*e*. the time between inoculation of carcinoma cells and appearance of palpable tumors (Fig 1C). There was furthermore no difference in time of reaching the designated harvest size (Fig 1D). External volumetric measurement of tumor sizes showed that tumor growth rates were not significantly different in wild-type and *Fmod*-/- mice (Fig 1E).

### Vascular and cellular composition is not affected by fibromodullin in OOC38 carcinoma

**Fig 2:**
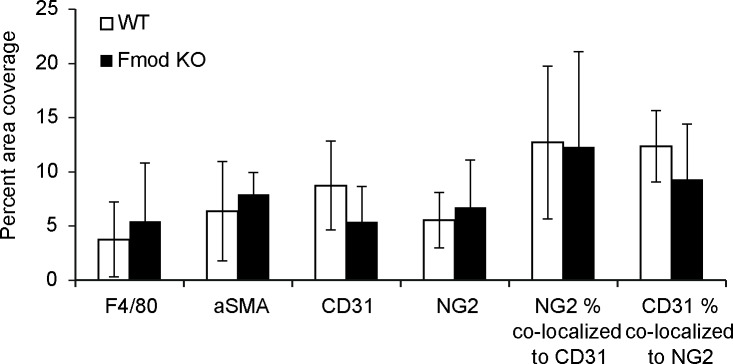
Cellular composition of tumors. Immunofluorescence images of OOC38-derived tumors grown in wild-type (n = 5) and *Fmod-/-* (n = 5) mice were used. Each of the markers was quantified as percentage of pixels per total number of pixels. Co-localization of NG2 and CD31 was quantified as percentage of co-localized pixels per total number of pixels. No differences between WT and *Fmod*-/- mice were observed (Mann-Whitney and Student’s *t*-test, p>0.05; error bars are standard deviations).

OOC38 carcinomas grown in wild-type and in *Fmod*-/- did not differ with regard to density of CD31-positive vessels nor with regard to vessel coverage by NG2-positive pericytes (Fig 2). Furthermore, no difference in the amounts of F4/80-positive macrophages or α-SMA-positive stromal cells could be detected (Fig 2). These data are in agreement with previous findings showing no difference in the cellular and vascular composition in KAT-4 carcinoma grown in wild-type or *Fmod*-/- SCID mice [22]. Thus, cellular parameters were unaffected by fibromodulin deficiency or the presence of an intact adaptive immune system.

### Effects of Fmod deficiency on structure and content of stroma collagen

**Fig 3:**
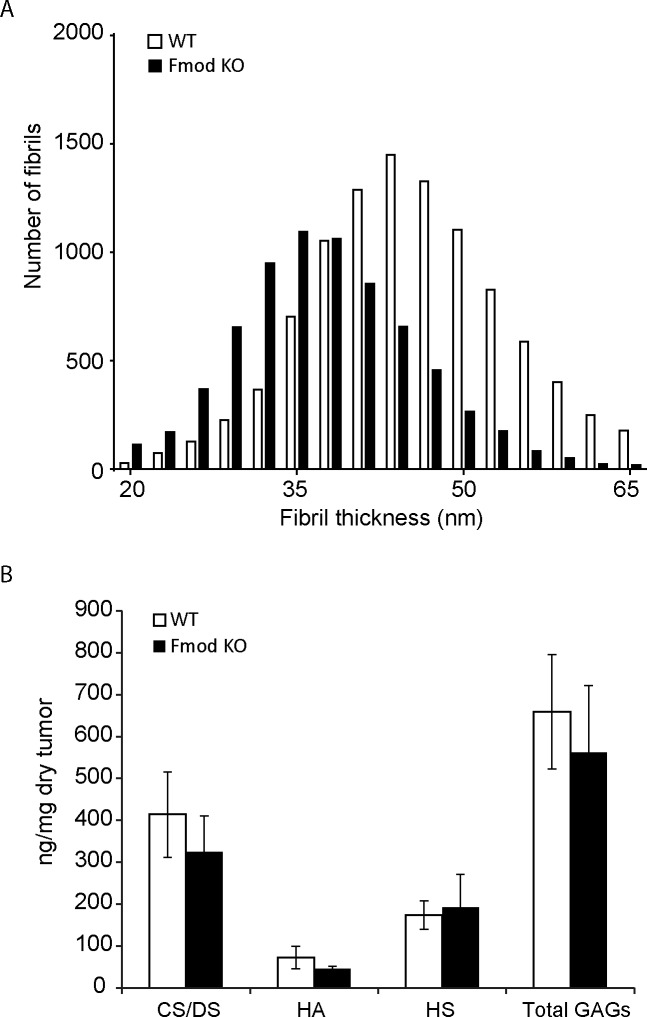
**Ultrastructural and content comparison of collagen and GAG composition** A. Histogram of collagen fibril diameter in OOC38 tumors measured in electron microscopic images (WT n = 10291, *Fmod*-/- n = 7119; Kolmogorov-Smirnov test p < 0.0001). Average collagen fibril diameter was: WT 44.8 nm (n = 4), *Fmod*-/- 37.8 nm (n = 5), Student’s *t* test p = 0.004. B. Total Chondroitin/Dermatan sulfate (CS/DS), Hyaluronic acid (HA), Heparan Sulfate (HS) and total glycosaminoglycan content (Total GAGs) represented in ng/mg of tumor by dry weight (WT n = 4, Fmod KO n = 4).

Treatment of KAT-4 experimental carcinoma with Imatinib increases perfusability of the carcinoma concomitant with a lowering of the mean collagen fibril diameter in the stroma [12]. KAT-4 carcinoma grown in *Fmod*-/- SCID mice had a similar phenotype, manifested in increased ECV, lowered IFP, and decreased collagen fibril thickness [22]. Based on these previous results we investigated whether fibromodulin deficiency decreased average collagen fibril thickness also in syngeneic OOC38 carcinoma grown in *Fmod*-/- C57BL6 mice. The distribution of collagen fibril diameters was shifted towards thinner fibrils in carcinoma grown in *Fmod*-/- compared to carcinoma grown in wild-type mice (Fig 3A). The average collagen fibril diameter was 37.8 ± 2.3 nm in *Fmod*-/- mice (n = 4) and in 44.8 ± 2.2 nm in wild-type mice (n = 4, p < 0.005).

### Effects of fibromodulin deficiency on the content and structure of glycosaminoglycans

Glycosaminoglycans and collagen network play substantial roles in fluid retention and hydraulic conductivity in tissues [23]. The hyaluronan (HA) and proteoglycan-rich ground substance provides the swelling pressure in loose connective tissues [23, 24]. Since we previously have established that fibromodulin-deficiency alters hydraulic properties of experimental carcinoma [22] it was important to investigate whether fibromodulin-deficiency alters the amount and composition of the HA and proteoglycan-rich ground substance. We therefore conducted structural and quantitative analysis of glycosaminoglycans in OO38 carcinoma grown in wild-type and *Fmod*-/- mice. Chondroitin sulfate/dermatan sulfate (CS/DS) and hyaluronan (HA), were slightly decreased in *Fmod*-/- tumors, without reaching statistical significance (p > 0.05) (CS/DS: wild-type = 414 μg/ mg dry tumor, sd = 102, *Fmod*-/- 325, sd = 86); HA: wild-type 72 μ g/ mg dry tumor, sd = 26, *Fmod*-/- 45, sd = 6) while heparan sulfate (HS) was unchanged (wild-type = 173 μg/ mg dry tumor, sd = 34, *Fmod*-/- 192, sd = 79) (Fig 3B). Likewise, the structure of the CS/DS and HS chains was unchanged (Table 2).

**Table 2:** Glycosaminoglycan (GAG) structure in OOC38 carcinomas grown in wild type and Fmod -/- mice (n = 4 + 4).

**CS/DS composition**				
	**WT**		**Fmod KO**	
	**average**	**Sd**	**average**	**Sd**
	(mol %)		(mol %)	
deltaUA-2S-GalNAc-4S(B)	1.21	0.42	1.79	0.95
deltaUA-GalNAc-4S-6S (E)	1.92	0.59	2.65	0.62
deltaUA-GalNAc-4S (A)	83.03	0.79	80.15	4.078
deltaUA-GalNAc-6S (C)	1.16	0.22	1.53	0.23
deltaUA-GalNAc (O)	12.68	1.19	13.89	3.65
**HS composition**				
	**WT**		**Fmod KO**	
	**average**	**Sd**	**average**	**Sd**
	(mol %)		(mol %)	
deltaUA-2S-GlcNS-6S	5.29	1.41	7.02	0.21
deltaUA-GlcNS-6S	7.16	0.560	8.40	0.61
deltaUA-2S-GlcNS	3.10	0.82	2.95	0.36
deltaUA-GlcNS	16.92	0.46	15.82	0.57
deltaUA,2S-GlcNAc,6S	2.12	0.49	1.95	0.35
deltaUA-GlcNAc-6S	13.08	0.99	13.36	0.51
deltaUA,2S-GlcNAc	0.00		0.00	
deltaUA-GlcNAc	52.34	2.15	50.50	0.83

## Discussion

Here we report that although the collagen network architecture is changed, neither the quantitative or qualitative properties of glycosmaminoglycans are affected by fibromodulin-deficiency, in a syngeneic experimental model of carcinoma. This finding is important in view of the established and intricate relationship between glycosaminoglycans and collagen networks in the control of interstitial fluid homeostasis [23, 24, 32, 33]. Our present data add to our previously published results, which show that fibromodulin-deficiency increases the extracellular fluid volume and lowers the interstitial fluid pressure in experimental carcinoma concomitant with a reduction of the collagen network density and collagen fibril thickness [22]. It is therefore possible to infer that fibromodulin-directed collagen network assembly can modulate the hydraulic properties of a carcinoma interstitium independently of glycosaminoglycans. The present data are in line with studies on the effects of Imatinib on experimental carcinoma [10–12, 14]. Treatment of collagen-rich experimental carcinoma with Imatinib lowers interstitial fluid pressure and increases blood flow, extracellular volume and fluid transport through the tumor interstitium, as well as reducing stromal collagen fibril thickness while leaving the glycosaminoglycans unaffected. Importantly, Imatinib also increases the uptake of and efficacy of chemotherapy [34, 35]. By its effects on collagen network assembly fibromodulin therefore constitutes a potential target for new treatments aimed to increase effectiveness of conventional anti-cancer treatment regimes. It should be emphasized that experimental modulation of glycosamonoglycans in carcinoma, such as treatment with hyaluronidase in models of pancreatic ductal adenocarcinoma (PDAC) also increases the exchange between the blood stream and tumor interstitium and, moreover, increases the efficacy of chemotherapy [2]. Thus, work in experimental models suggest that modulations of tumor ECM to increase uptake of drugs can target both or any of the major structural components collagen or glycosaminoglycans.

Several reports have compared colon cancerous tissues with their normal counterparts with regard to proteoglycans and glycosaminoglycans. The amounts of CS/DS and HS decreased in cancer tissues and their structures differed in a limited way [36]. Another report showed a dramatic increase of decorin and versican in colon adenocarcinoma, which constitute the vast majority of the CS/DS-proteoglycans, and a deep altered chain composition and length [37]. A comparison of our results with these previous results is obviously inapplicable due to different tumor histological aspects. Here we report on unaltered levels of glycosaminonglycans in OOC38 carcinoma grown in wild-type and *Fmod -/-* mice. OOC38 carcinoma are highly cellularized and it seems reasonable to assume that HS is mostly produced by the malignant cells. Similar HS content and structure (Table 2) could reflect similar densities of malignant cells in wild-type and *Fmod -/-* mice in line with the finding that growth characteristics in the two genotypes were unaltered. Previous studies have shown that in colon carcinoma HA is mainly expressed in the connective tissue of the stroma, while the tumor parenchyma is mostly devoid of HS [38]. The structure of CS/DS was similar in OOC38 carcinoma grown in wild-type and *Fmod -/-* mice. This structure was dominated by the presence of 4-O-sulfated N-acetyl-galactosamine (80–83%), with much less abundant not-sulfated (13–14%), and 6-O-sulfated (1–2%) moieties. The sum of the di-sulfated structures was 3–4%. This composition is similar to CS/DS chains found in the most common interstitial proteoglycans in carcinoma, compatible with the recently described onco-fetal CS found on virtually all malignant cells and in cancer tissues [39].

The mechanism behind the collagen fibril phenotype seen in carcinoma grown in *Fmod -/-* mice could be related to an impaired lysyl oxidase-driven collagen cross-linking given the fact that fibromodulin interacts with lysyl oxidases [40, 41], compounded by a dysregulated, otherwise fibromodulin-modulated, collagen fibrillogenesis. The structural imperfections in collagen fibrils could expose them to MMPs (collagenases) that would digest the fibrils into thinner structures [40]. The same scenario could result from the lack of fibromodulin inhibiting MMPs through binding to MMP cleavage site [40–42].

Fibromdulin reportedly stimulates angiogenesis [43] but our present data in OOC38 and previous studies in KAT-4 carcinoma [22] did not show any difference in the presence of CD31-positive vessels in carcinoma grown in wild-type or *Fmod -/-*. Furthermore, other investigated cellular markers were not altered in carcinoma grown in mice of the two genotypes, suggesting that the role of fibromodulin in carcinoma may not be pivotal in aspects not related to collagen.

Overall, the question of the role of fibromodulin in tumor stroma is of potential clinical relevance as it affects the physiological barrier in solid tumors. Our data suggest an important role of fibromodulin in the modulation of collagen fibrils in tumor stroma, and thus a role in accessibility of anti-cancer of drugs.
